# Analysis of energy loss in the helical hedge flow channel of fruit tree root emitter

**DOI:** 10.1038/s41598-023-49035-y

**Published:** 2023-12-07

**Authors:** Jun Zhang, Xu Li, Shouping Zhang, Mengli Zhang

**Affiliations:** Chongqing Water Resources and Electric Engineering College, Yongchuan, 402160 Chongqing China

**Keywords:** Fluid dynamics, Agroecology

## Abstract

This paper proposes the design of a helical hedge flow channel with a high energy loss, which shows promising potential for application in fruit tree root emitters. The aim is to investigate the relationship between the energy loss form in the channel and its influencing factors. The hydraulic performance testing method is employed to analyze the factors that affect energy loss. The main influencing factors are determined using the response surface methodology (RSM) for experimental design. Based on the obtained experimental results, the energy loss form and influencing factors are analyzed, and a prediction model for the energy loss coefficient (*ξ*) is established. The results indicate that the *ξ* exhibits a decreasing trend with an increase in the diversion angle (*α*), a trend of first increasing and then decreasing with an increase in the channel width (*b*), and an increasing trend with an increase in the number of channel units (*n*). The effects of the straight section length (*l*_1_), convergence section length (*l*_2_), and bend radius (*r*) on the *ξ* can be neglected. The ranking of the geometric parameters' influence on the *ξ* is as follows: *n* > *b* > *α* > *l*_1_ > *r* > *l*_2_. The experimental results reveal that the *ξ* ranges from 19.2 to 234.3. Furthermore, the head loss along the flow channel constitutes merely 0.06–0.47% of the local head loss, The main form of energy loss in the spiral counter flow channel is local head loss. There is a significant linear relationship between *α*, *b*, *n* and the *ξ*, The established prediction model (R^2^ = 0.9691) can accurately predict the *ξ* of the channel.

## Introduction

Subsurface irrigation is an efficient water-saving irrigation method where water can be directly delivered to the root zone of plants, meeting their growth requirements. This approach helps avoid water waste and loss on the soil surface, thereby improving water utilization efficiency ^1–3^. In recent years, researchers have proposed various novel subsurface irrigation technologies, such as subsurface drip irrigation ^4,5^, spring gushing root irrigation ^6,7^, porous ceramic pipe irrigation ^8,9^, and micro-sprinkler irrigation ^10,11^. Scholars often evaluate the performance of emitter based on criteria such as energy loss ^12^, hydraulic performance ^13^, and clogging resistance ^14^. Different subsurface emitter have different outlet forms and design flow rates, which correspond to different channel configurations. The channel configuration of an emitter directly determines its irrigation effectiveness and water utilization efficiency. Typically, by analyzing the factors affecting energy loss in the channel, researchers further optimize the channel structure to improve the flow performance of the emitter.

The energy loss of water flow inside the emitter channel is a critical factor that affects flow stability. Understanding the relationship between channel parameters and energy loss is crucial for optimizing channel structure and improving hydraulic performance. Energy dissipation is typically analyzed by observing changes in inlet and outlet pressure and flow rate^15,16^, with the energy loss coefficient (ξ) serving as an indicator of the channel's ability to dissipate water flow energy. Zhang et al. employed PIV technology to investigate energy loss in labyrinth emitter channels and discovered the correlation between Reynolds number and head loss, making significant contributions to the subsequent optimization of labyrinth irrigation channels^17^. Xu et al. used CFD methods to simulate emitter channels, enabling the development of numerical simulation techniques suitable for different channel types^18,19^. This approach provides an efficient research method for studying energy loss in channels. Previous studies have examined water flow energy loss under various irrigation parameters using macroscopic experimental methods, while PIV technology and numerical simulations have been employed to analyze the microscopic mechanisms that cause changes in flow fields and energy loss. The research findings obtained through experimental and numerical simulation methods offer valuable data support for optimizing and designing different types of emitter channels.

**Figure 1:**
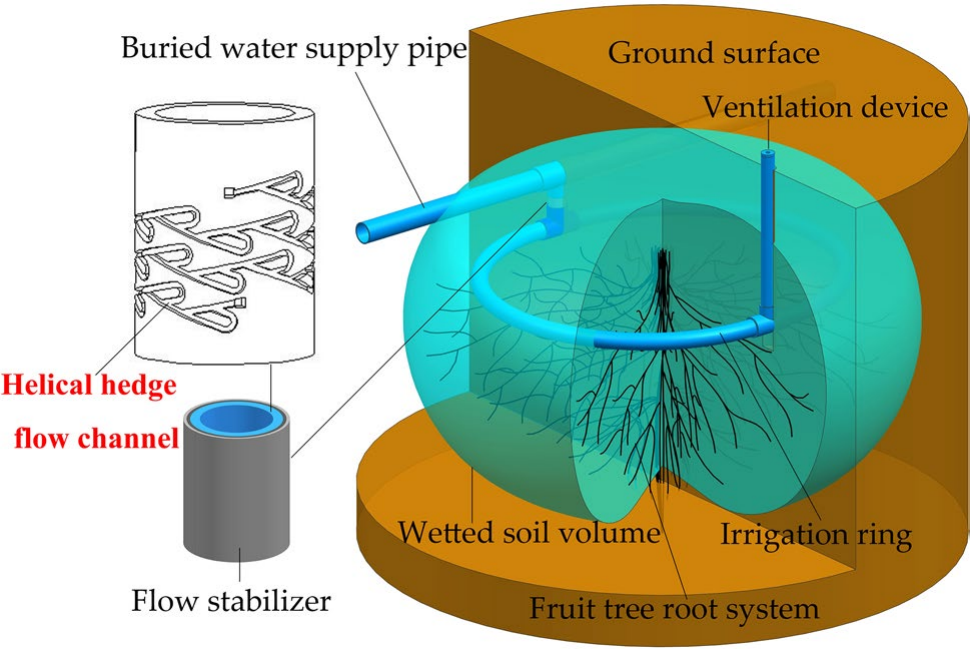
Fruit tree root emitters.

Studies have indicated that subsurface circular irrigation is highly effective for crops in extremely arid regions^20^. To further enhance irrigation water use efficiency and ensure that the underground soil moisture zone aligns with the root system of fruit trees, we propose a novel Fruit tree root emitters (Fig. 1). In order to achieve a balanced flow rate for different high-pressure water streams passing through the emitter, this article designs a helical hedge flow channel at the inlet of the fruit tree root emitter using the principle of effective pressure reduction and energy loss of hydrogen gas through a Tesla pressure relief valve^21^. Preliminary experimental results have shown that when this type of flow channel is applied to an emitter, the flow rate does not continuously increase with increasing pressure, but instead remains within a relatively stable range, achieving a high level of steady flow. This indicates its high potential in the field of emitter applications. To investigate the steady flow principle of this flow channel, a series of indoor experiments were conducted using the single-factor and response surface experimental methods. The study achieved the following: (1) identification of the main geometric parameters that affect the energy loss coefficient of the flow channel, (2) identification of the primary forms causing energy loss in the flow channel and (3) establish and evaluate a predictive model for the energy loss coefficient. The research results can provide reliable data support and serve as a reference for designing and optimizing this type of flow channel in various emitters.

## Methods used

### Working principle of fruit tree root emitter

The effectiveness of irrigation water usage is directly determined by how well the soil moisture profile matches with the root system of fruit trees after irrigation. To prevent losses from surface evaporation and deep percolation, and to create a ring-shaped soil moisture profile that aligns with the root zone of fruit trees after irrigation, we have developed a novel buried ring-shaped root irrigation device specifically designed for fruit trees. Figure 1 illustrates the working principle of the root emitter. One root emitter is pre-buried near the root system of each fruit tree. The root emitter consists of flow stabilizer, irrigation ring, and ventilation device. Within the buried water supply pipeline, the water flow within a certain pressure range passes through the flow stabilizer of each fruit tree root emitter. It then enters the irrigation ring with a relatively stable flow rate. Through a certain number of outlet holes in the irrigation ring, the water slowly infiltrates into the soil. The ventilation device helps eliminate the clogging of outlet holes caused by negative pressure in the irrigation ring. After irrigation, a surrounding soil moisture zone is formed with the root system of the fruit tree at the center.

**Figure 2:**
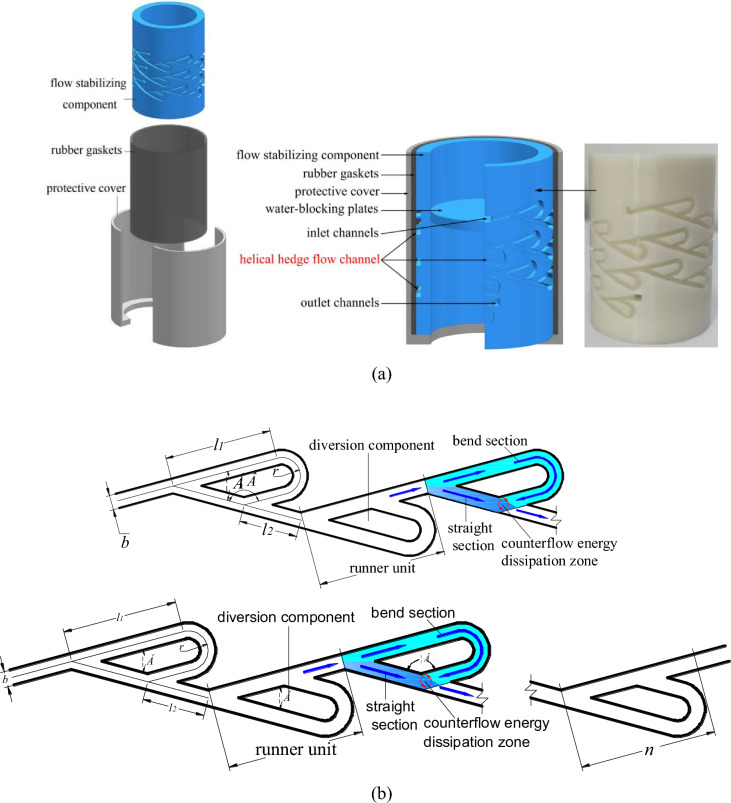
Structure and working principle of flow stabilizer (**a**) 3D model (**b**) flow channel layout.

The flow stabilizer utilizes a novel design of a helical hedge flow channel (Fig. 2), which bidirectional diversion, rapid counter-current, energy dissipation, and flow stabilization. Figure 2a is a physical representation of the 3D cross-section and the flow stabilizing component of the flow stabilizer. The flow stabilizer is composed of three main components from the inside out: the flow stabilizing component, rubber gaskets, and two protective covers. The flow stabilizing component consists of water-blocking plates, inlet channels, helical hedge flow channel, and inlet and outlet channels. The outer layer of the rubber gaskets is surrounded by two symmetrical movable protective covers. When the outer walls of the protective covers at the upper and lower ends are connected to the inner walls of the water supply tee and the irrigation water ring tee, the protective covers tightly enclose the rubber gaskets. The outer wall of the flow stabilizing component is equipped with a certain number of inward-facing counter flow channels arranged along a spiral line. After the water flows through the helical hedge flow channel, it exits through the outlet, enters the bottom of the flow stabilizing component, and eventually enters the irrigation ring.

The schematic diagram of the helical hedge flow channel and the geometric parameters are shown in Fig. 2b. Each flow channel unit is defined as the channel between the diversion starting section and the confluence ending section. The geometric parameters of each flow channel unit include the channel width (*b*), the length of the straight section of the bend (*l*_1_), the radius of the bend (*r*), the diversion angle (*α*), the counter flow angle (*β*), where the *β* is complementary to the *α*, and the length of the confluence section (*l*_2_). The number of runner units is represented by "*n*". Within each unit, the flow is diverted to the bend and the straight section under the action of the diversion component. The flow undergoes a sharp turn in the bend and counter flows with the flow in the straight section in the energy dissipation zone. The flow separation and confluence in the channel section result in local head losses, which dissipate a large amount of kinetic energy. Through progressive energy dissipation, a stable flow pattern is achieved.

### Single-factor experimental plan

In order to investigate the influence of various parameters of the helical hedge flow channel on the energy loss coefficient, a single-factor experiment is conducted with each channel parameter as an independent variable. As shown in Fig. 2, when the outer diameter of the cylindrical flow stabilizer is constant, a unique helical hedge flow channel can be determined by the parameters *α*, *b*, *n*, *l*_1_, *l*_2_, and *r*. Therefore, in the single-factor experiment, these six parameters are taken as independent variables to explore the degree of influence of each factor on the hydraulic performance of the channel. The outer diameter of the flow stabilizer in this experiment is 28 mm, and preliminary exploratory experiments are conducted within the pressure range of 50–250 kPa. The results show that when 30° < *α* < 60°, 0.5 mm < *b* < 2.5 mm, 10 < *n* < 50, 5 mm < *l*_1_ < 13 mm, 2 mm < *l*_2_ < 10 mm, and 0.75 mm < *r* < 1.75 mm, the flow rate is close to the design flow rate. Therefore, this range is taken as the value range for the single-factor experiment. Within this value range, each parameter is set at 5 equidistant levels. The single-factor experimental method is used, assuming that there is no interaction between the parameters. When one parameter is changed, the other parameters remain at their intermediate level values for the experiment. The experimental plan is shown in Table 1.

**Table 1:** Single-factor experimental plan.

Factors		*α*	*b* (mm)	*n*	*l*_1_ (mm)	*l*_2_ (mm)	*r* (mm)
Levels	1	30°	0.5	10	5	2	0.75
	2	37.5°	1	20	7	4	1
	3	45°	1.5	30	9	6	1.25
	4	52.5°	2	40	11	8	1.5
	5	60°	2.5	50	13	10	1.75

### Response surface experimental design

Based on the results of the single-factor experiment, the main parameters *α*, *b*, and *n* that affect the energy loss coefficient are selected as factors for the response surface experiment. When *α*, *b*, and *n* are within the range of 30° < *α* < 60°, 1 mm < *b* < 2 mm, and 10 < *n* < 40, and the inlet pressure is within 50–250 kPa, the flow values corresponding to different pressures are close to the design flow. Therefore, this range is chosen as the range for the levels of each factor in the response surface experiment. Three levels are set within this range, and the designed factors and levels for the response surface experiment are shown in Table 2.

**Table 2:** Factors and levels for the response surface experiment.

Factors		*α*	*b* (mm)	*n*
Levels	− 1	30°	1	10
	0	45°	1.5	25
	1	60°	2	40

To scientifically reduce the experimental workload and fully consider the random errors in the experiments, and to ensure that the analysis results are conducive to seeking the optimal values, the Box-Behnken Design in Design Expert 11 software was chosen to design the response surface experimental plan based on the factors and levels presented in Table 2. The software utilized factors *α*, *b*, and *n*, with the *ξ* as the response variable, resulting in a total of 17 experimental plans. The specific experimental plan is shown in Table 3.

**Table 3:** Response surface experimental design.

Experimental plan	Factors and levels			Experimental plan	Factors and levels		
	*α*	*b*	*n*		*α*	*b*	*n*
1	0	0	0	10	0	1	1
2	− 1	0	− 1	11	0	0	0
3	1	0	− 1	12	− 1	1	0
4	0	0	0	13	1	− 1	0
5	− 1	− 1	0	14	1	1	0
6	− 1	0	1	15	0	0	0
7	0	− 1	1	16	0	1	− 1
8	0	0	0	17	1	0	1
9	0	− 1	− 1				

### Experimental setup

**Figure 3:**
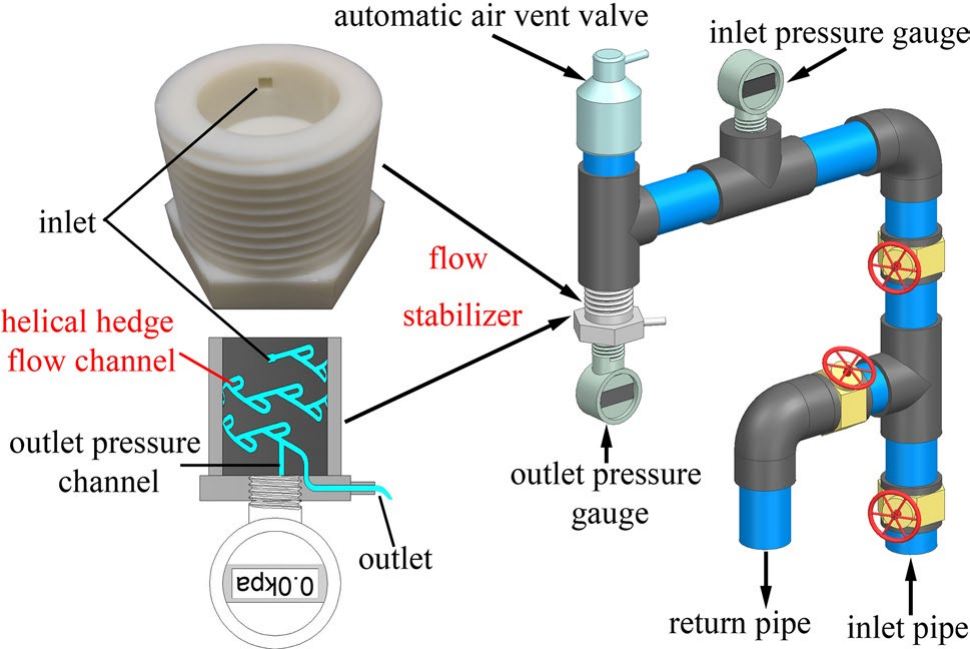
Experimental setup.

The helical hedge flow channel was modeled in 2D and 3D using AutoCAD and Unigraphics NX software. A 3D printer with a precision of 0.1 mm was used to fabricate the prototypes. To ensure experimental accuracy and eliminate errors caused by the properties of rubber gaskets, the flow stabilizer's flow stabilizing components, rubber gaskets, and protective covers were 3D printed using PLA material. External and internal threads were generated at the upper and lower ends, respectively, to connect the stabilizer with the water supply tee and the outlet pressure gauge with threaded connections. The experimental setup is illustrated in Fig. 3. Digital pressure gauges with an accuracy of 0.01 kPa were installed upstream and downstream of the stabilizer. To eliminate the interference of gas in the pipeline on the flow, an automatic air vent valve was installed at the upper end of the inlet. The water supply channels were constructed using PVC pipes with an outer diameter of 32 mm and corresponding fittings to ensure airtight connections.

### Detailed methodology

The quality of a emitter is determined by the uniformity of the outflow rate of water passing through its flow channel within a specific pressure range. This hydraulic performance is typically evaluated through indoor testing. In accordance with this principle, a self-circulating test device (Fig. 3) was designed for conducting this experiment. The water used for the experiment is taken from the laboratory tap water. The water temperature is kept constant at 23 °C for each test. Water is supplied to the device through a pressure-adjusting water supply pipeline. The variable frequency pump is adjusted to achieve the required inlet pressure. To eliminate any interference caused by gas during the experiment, an automatic exhaust valve is installed at the top of the system. This valve allows the gas to be automatically discharged after the water passes through the inlet pressure gauge. Then the water flows into the helical hedge flow channel through the inlet of the flow regulator. Finally, after filling the outlet pressure channel, the water flows out from the outlet duct at the end of the flow channel. After the operation stabilizes, the outlet pressure is read, and the flow rate is measured using a stopwatch and a measuring cylinder. According to the specifications and test methods of GB/T17187-2009/ISO9261:2004 "Agricultural irrigation equipment–Emitters and emitting pipe-Specification and test methods," the outlet flow rate and pressure are tested under different inlet pressure of 50, 75, 100, 125, 150, 175, 200, 225, 250 kPa. Three sets of 3D printed flow regulator components are made for each scheme, and each flow regulator component undergoes three sets of repeated tests. When the relative error of the repeated test results for the same scheme is less than 3%, the average value is taken as the test result.

**Figure 4:**
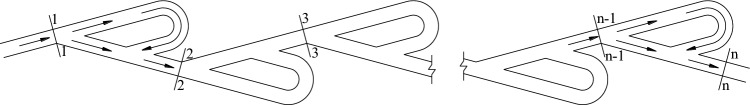
Schematic diagram of flow in the flow channel of the flow stabilizer.

Research has shown that increasing head loss can effectively improve the hydraulic performance of emitters ^22^. Therefore, it is of great significance to understand the relationship between the flow channel parameters of the flow regulator and energy loss in order to enhance its flow stability. The new helical hedge flow channel of this flow regulator does not have an accurate method for calculating energy loss. Since the cross-section of the flow channel is square, it can be approximated as a rectangular channel. Therefore, the calculation method for head loss in rectangular pipes in hydraulic theory is used as a reference to approximately calculate the total head loss in the helical hedge flow channel. The flow in the helical hedge flow channel is shown in Fig. 4, with arrows indicating the direction of water flow. At the inlet of each flow channel unit, a cross-section is selected, resulting in a total of n cross-sections for the n flow channel units in the entire flow channel.

From Fig. 4, it can be observed that when the water flows through two adjacent sections, it undergoes branching, sharp turns, and collisions, resulting in energy loss. Assuming that the flow between the adjacent water sections in the flow channel of Fig. 4 satisfies the Bernoulli equation, we can apply the Bernoulli equation to each adjacent section.

1 $$\begin{gathered} \frac{{P_{1} }}{\rho g} + \frac{{v_{1}^{2} }}{2g} + z_{1} = \frac{{P_{2} }}{\rho g} + \frac{{v_{2}^{2} }}{2g} + z_{2} + \xi_{1} \frac{{v_{2}^{2} }}{2g} + \lambda \cdot \frac{{l_{1} }}{4R} \cdot \frac{{v_{2}^{2} }}{2g} \hfill \\ ...... \hfill \\ \frac{{P_{n - 1} }}{\rho g} + \frac{{v_{n - 1}^{2} }}{2g} + z_{n - 1} = \frac{{P_{n} }}{\rho g} + \frac{{v_{n}^{2} }}{2g} + z_{n} + \xi_{n - 1} \frac{{v_{n}^{2} }}{2g} + \lambda \cdot \frac{{l_{n - 1} }}{4R} \cdot \frac{{v_{n}^{2} }}{2g} \hfill \\ \end{gathered}$$

where *P*_*i*_ is the average pressure at the *i*-th section, Pa; *v*_*i*_ is the average flow velocity at the *i*-th section, m/s; ρ is the density of the liquid, g/cm^3^; g is the acceleration due to gravity, m/s^2^; *ξ*_*i*_ is the local head loss coefficient from section *i* to section *i* + 1; *λ* is the head loss coefficient along the channel; *l*_*i*_ is the length of the flow channel from section *i* to section *i* + 1, m.

According to the continuity equation, it can be known that:

2 $$Q = v_{1} A_{1} = v_{2} A_{2} { = }v_{3} A_{3} { = } \cdot \cdot \cdot { = }v_{{\text{n}}} A_{n}$$

Besides:

3 $$A_{1} = A_{2} { = }A_{3} { = } \cdot \cdot \cdot { = }A_{n}$$

From Eqs. (2) and (3), it can be deduced that:

4 $$v_{1} = v_{2} { = }v_{3} { = } \cdot \cdot \cdot { = }v_{{\text{n}}} = v$$

By substituting Eq. (4) into each equation of Eq. (1), adding them together and rearranging, we obtain:

5 $$\frac{{P_{1} - P_{n} }}{\rho g} = z_{n} - z_{1} + \left( {\xi_{1} + \xi_{2} + \cdot \cdot \cdot + \xi_{n - 1} } \right)\frac{{v^{2} }}{2g} + \lambda \frac{{\left( {l_{1} + l_{2} + \cdot \cdot \cdot + l_{n} } \right)}}{4R} \cdot \frac{{v^{2} }}{2g}$$

Define:

6 $$\begin{gathered} \Delta P = P_{1} - P_{n} \hfill \\ L = l_{1} + l_{2} + \cdot \cdot \cdot + l_{n} \hfill \\ \end{gathered}$$

The estimated magnitude of the relative head loss due to the difference in water head between the inlet and outlet positions is approximately 10^–4^, which can be neglected, i.e.,

7 $$z_{n} - z_{1} \approx 0$$

Substituting Eqs. (6) and (7) into Eq. (5), we obtain:

8 $$\frac{\Delta P}{{\rho g}} = \left( {\xi_{1} + \xi_{2} + \cdot \cdot \cdot + \xi_{n - 1} + \lambda \frac{L}{4R}} \right)\frac{{v^{2} }}{2g}$$

If we define the energy loss coefficient (*ξ*) of the channel as:

9 $$\xi = \xi_{1} + \xi_{2} + \cdot \cdot \cdot + \xi_{n - 1} + \lambda \frac{L}{4R}$$

Then Eq. (8) can be simplified as:

10 $$\frac{\Delta P}{{\rho g}} = \xi \frac{{v^{2} }}{2g}$$

The energy loss coefficient *ξ* can be expressed as:

11 $$\xi = 2\frac{\Delta P}{{v^{2} \rho }}$$

Alternatively, it can be expressed in terms of flow rate:

12 $$\xi = 2\frac{{\Delta P \cdot b^{4} }}{{Q^{2} \rho }}$$

Equation (12) can also be expressed as:

13 $$Q = b^{2} \sqrt {\frac{2\Delta P}{{\rho \cdot \xi }}}$$

The total head loss generated by the flow in the helical counterflow channel includes the head loss along the path and the local head loss. According to the superposition theory of energy losses, the total head loss can be obtained as:

14 $$h_{w} = h_{f} + h_{j}$$

Thus, the total head loss can be calculated using the following equation:

15 $$h_{w} = \xi \frac{{v^{2} }}{2g}$$

where the energy loss coefficient *ξ* can be calculated using Eq. (12). According to the Darcy–Weisbach formula, the head loss along the path is:

16 $$h_{f} = \lambda \cdot \frac{L}{4R} \cdot \frac{{v^{2} }}{2g}$$

The cross-section of the channel is square, and the hydraulic radius formula for rectangular sections can be used to obtain:

17 $$R = \frac{A}{\chi } = \frac{{b^{2} }}{4b} = \frac{b}{4}$$

In the turbulent smooth zone of the irrigation channel, the head loss coefficient along the path is often calculated using the Blasius formula:

18 $$\lambda = \frac{0.3164}{{Re^{0.25} }}$$

where the Reynolds number is defined as:

19 $$Re = \frac{vR}{\upsilon }$$

where *h*_*w*_ is the total head loss, m; *h*_*f*_ is the head loss along the path, m; *h*_*j*_ is the local head loss, m; *λ* is the head loss coefficient along the path; *l* is the channel length, m; *R* is the hydraulic radius, m; *χ* is the wet perimeter, m; *Re* is the Reynolds number; $\upsilon$ is the kinematic viscosity, cm^2^/s (The water temperature in this experiment is 23 °C, so $\upsilon$ = 0.0094 cm^2^/s). *A* is the cross-sectional area of the confluence section, m^2^; *v* is the average flow velocity in the confluence section, m/s; *L* is the length of the channel center line, m; *ξ* is the energy loss coefficient; *∆P* is the pressure difference between the inlet and outlet, Pa; *Q* is the outlet flow rate, m^3^/s.

## Results and discussion

### The influence of single factors on the energy loss coefficient

**Figure 5:**
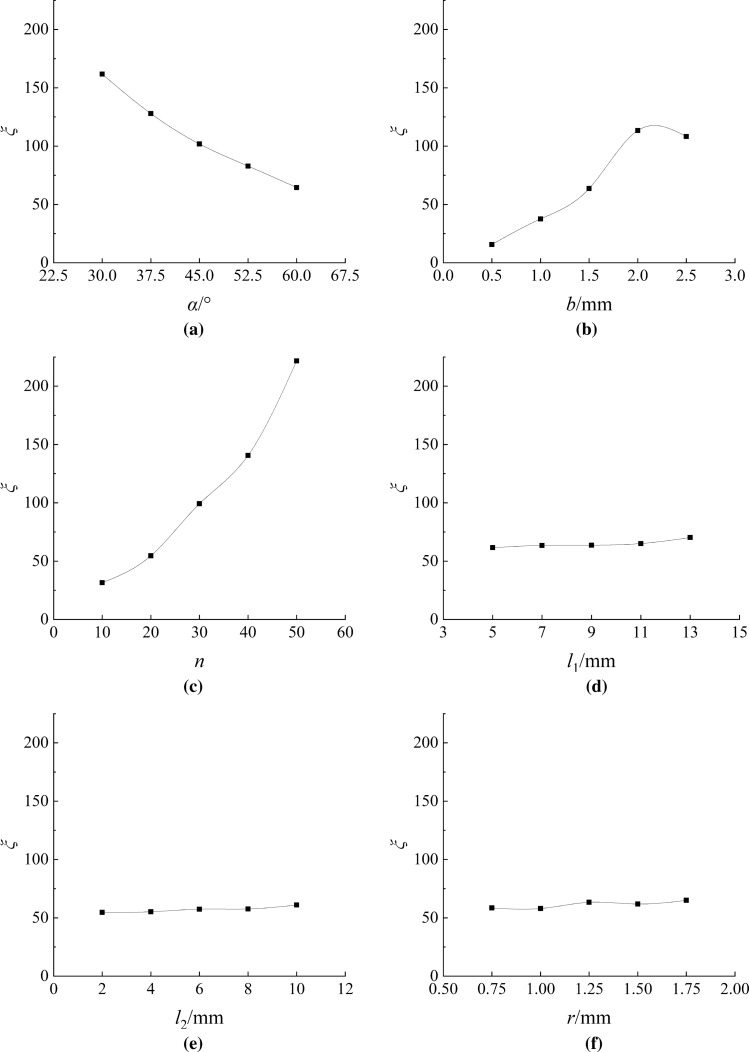
The influence of single factors on the energy loss coefficient (**a**) *α* (**b**) *b* (**c**) *n* (**d**) *l*_1_ (**e**) *l*_2_ (**f**) *r.*

According to the experimental results and Eq. (12), the energy loss coefficients for different schemes under different inlet pressure conditions are calculated. From the calculation results, it can be observed that there is a small difference in the energy loss coefficients for the same channel scheme under different pressures, while there is a significant difference in the energy loss coefficients between different channel schemes. This indicates that the influence of pressure on the energy loss coefficient can be neglected, and the variation is mainly caused by the channel parameters. The mean value within the pressure range of each channel scheme is taken to represent the corresponding energy loss coefficient. Figure 5 shows the relationship between various geometric parameters and energy loss coefficients in the single-factor experiment.

Figure 5a clearly demonstrates that a smaller *α* results in a higher *ξ*. This is because as the *α* decreases, the angle between the curved section and the straight section at the end approaches a right angle. The water flow entering the beginning of the curved section increases due to the inertia of the flowing water before diversion. Consequently, it becomes easier for the flow at the end to enter the straight section, leading to the generation of reverse flow in the straight section. This reverse flow then re-enters the beginning of the curved section, resulting in increased energy loss. From Fig. 5b,c, it can be seen that the energy loss coefficient initially increases and then decreases with an increase in *b* and n. This is because each channel unit causes a certain amount of energy loss, and as the *n* increases, the cumulative energy loss also increases, leading to a higher energy loss coefficient. From Fig. 5d–f, it can be seen that the energy loss coefficient increases with an increase in *l*_1_, *l*_2_, and *r*, but the increase is relatively small compared to the effects of *α*, *b*, and *n* in Fig. 5a–c. This indicates that the main channel parameters that cause changes in the energy loss coefficient are *α*, *b*, and *n*. Based on the variations in the curves in Fig. 5a–c, the impact of these three geometric parameters on the energy loss coefficient can be ranked as follows: *n* > *b* > *α*. The main parameters that affect changes in the energy loss coefficient are *α*, *b*, and *n*. The effects of *l*_1_, *l*_2_, and *r* can be neglected. However, *l*_1_, *l*_2_, and *r* are crucial parameters that determine the length of the channel (i.e., the key parameters that cause changes in the head loss along the flow path). This indicates that the head loss along the flow path of the helical counter-flow channel can be considered negligible compared to the local head loss. Based on the results of the single-factor experiments, if we want to obtain a channel design with the maximum energy loss coefficient, the combination of geometric parameters that corresponds to the maximum energy loss coefficient is *α*_1_*b*_4_*n*_5_*l*_1*-*5_*l*_2*-*5_*r*_5_. Through range analysis, it can be determined that the ranking of the influence of geometric parameters on the energy loss coefficient is as follows: *n* > *b* > *α* > *l*_1_ > *r* > *l*_2_.

### The impact of interaction between main influencing factors on the energy loss coefficient

By substituting the response surface experimental results into Eqs. (14)–(16), it is calculated that the head loss along the channel for each scheme ranges from 0.04 to 0.94 m, while the local head loss ranges from 4.1 to 25.08 m. The head loss along the channel accounts for only 0.06–0.47% of the local head loss. This indicates that the main form of energy loss in the channel is local head loss, while the head loss along the channel can be neglected. The energy loss coefficient calculated falls within the range of 19.2–234.3.

**Figure 6:**
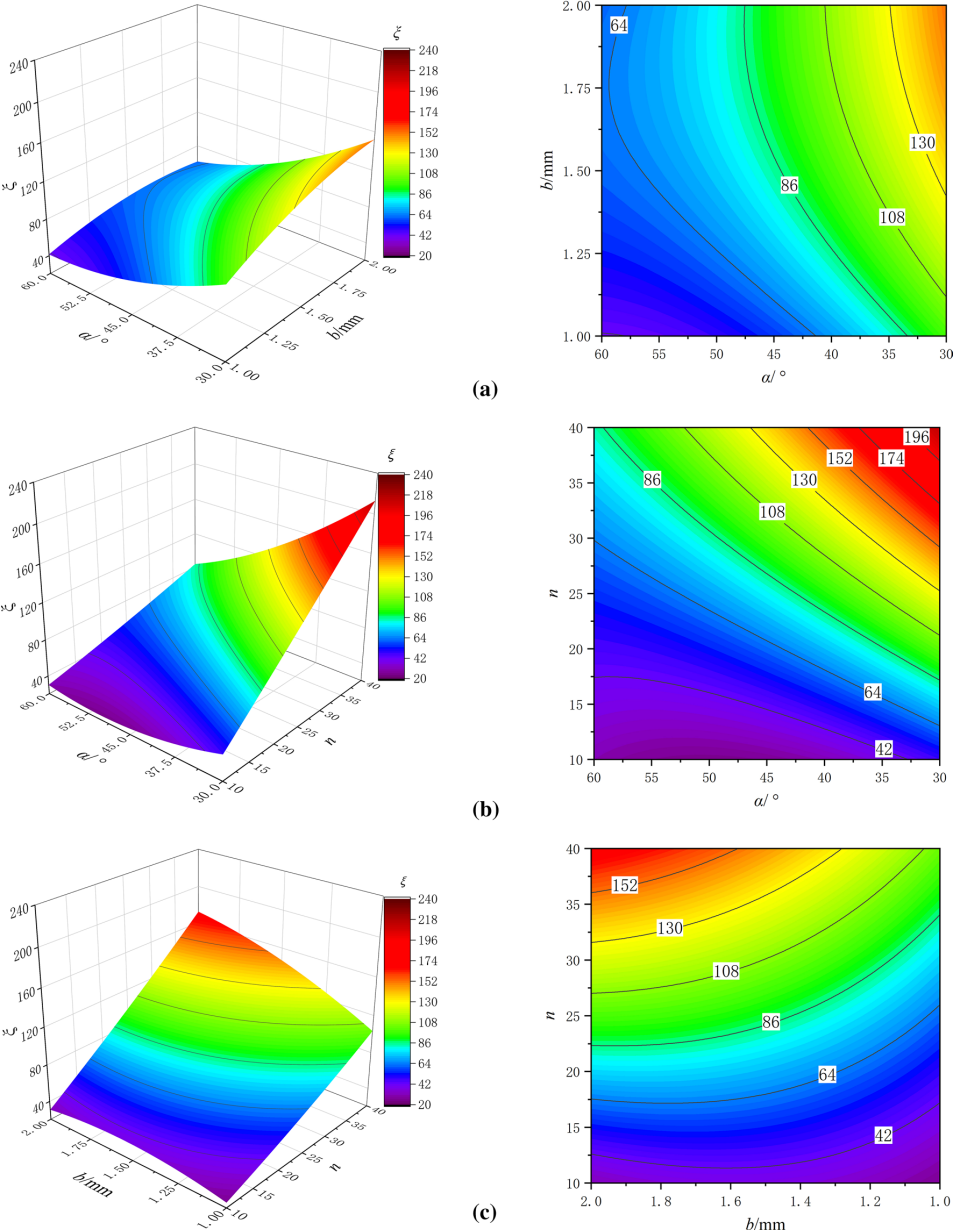
Response surface and contour plots of the energy loss coefficient (**a**) *α *&* b* (**b**) *α *&* n* (**c**) *b *&* n*.

The response surface experimental results were substituted into Eq. (12) to obtain the energy loss coefficient under different conditions. The response surface and contour plots of the energy loss coefficient were plotted, as shown in Fig. 6. From the three response surface plots in Fig. 6, it can be observed that the response surface is close to a plane, indicating a linear relationship between the *α*, *b*, *n*, and energy loss coefficient. In Fig. 6b, the response surface shows a large variation in both directions of the independent variables, and the energy loss coefficient exhibits significant differences with the variation of one independent variable when the other independent variable changes. This suggests that there is an interaction between the *α* and *n* in influencing the energy loss coefficient.

From Fig. 6a,b, it can be observed that the energy loss coefficient increases with a decrease in the *α*. This is because a smaller *α* leads to a closer angle of 180° between the curved section and the end of the straight section. Under the inertia effect of the incoming flow before diversion, more water flows into the beginning of the curved section, making it easier for the water to flow into the straight section, resulting in a reverse flow in the straight section. This reverse flow re-enters the beginning of the curved section, causing a secondary energy loss and an increase in the energy loss coefficient. From Fig. 6a,c, it can be observed that the energy loss coefficient increases with an increase in b, but the increase is relatively small. This is because with an increase in *b*, the flow capacity increases, and the energy loss caused by the collision of water molecules during sharp turns and flushing in the channel also increases, resulting in an increase in the energy loss coefficient.

From Fig. 6b,c, it can be observed that the energy loss coefficient shows the most significant increase with an increase in the n. This is because each level of channel unit causes a certain amount of energy loss in the flow. According to the principle of energy loss accumulation, the more channel units there are, the greater the energy loss, and the larger the corresponding energy loss coefficient of the channel. The rate of change of the three surface plots in the directions of the main channel parameters indicates that the order of the rate of change of the surface plots with respect to the independent variables is: *n* > *α* > *b*.

### Energy loss coefficient prediction model and analysis of variance

In order to more comprehensively predict the relationship between the energy loss coefficient and the channel parameters within the experimental boundary conditions, a multivariate regression analysis was conducted using Design Expert software, resulting in a quadratic regression model for *α*, *b*, *n*, and the energy loss coefficient.

20 $$\begin{gathered} \xi = - 56.08 - 2.82\alpha + 153.98b + 5.55n - 1.17\alpha b \hfill \\ - 0.12\alpha n + 2.12bn + 0.06\alpha^{2} - 39.13b^{2} + 0.01n^{2} \hfill \\ \end{gathered}$$

The variance analysis and regression coefficient significance test for the above regression models are shown in Table 4. The F values in the table are all significantly higher than the critical value F_0.01_, suggesting that Eq. (20) is highly significant.. From the P values in Table 4, it can be observed that the first-order terms of the energy loss coefficient are all extremely significant. Only the interaction term between the *α* and the *n* is extremely significant, while the remaining interaction terms and quadratic terms are not significant. This suggests that the main parameters have a significant linear relationship with the energy loss coefficient. Additionally, the interaction between the *α* and the *n* has a highly significant impact on the energy loss coefficient. This indicates that the energy loss coefficient is influenced by the *n* (*α*) as the *α* (*n*) changes. The coefficient of determination (*R*^2^) for the model is 0.9691, and the adjusted coefficient of determination (Adj *R*^2^) is 0.9294. The difference between the coefficient of determination (*R*^2^) and the adjusted coefficient of determination (Adj *R*^2^) is less than 0.2, indicating a good correlation between the dependent variable and the independent variables. This suggests that within the range of parameter values in this experiment, Eq. (20) can be used to predict the energy loss coefficient.

**Table 4:** Variance analysis of the response surface for the energy loss coefficient.

Source	Sum of squares	Degrees of freedom	Mean square	F value	P value
Model	43,111.03	9	4790.11	24.42	0.0002**
A-*α*	10,828.11	1	10,828.11	55.19	0.0001**
B-*b*	2738.14	1	2738.14	13.96	0.0073**
C-*n*	24,029.5	1	24,029.5	122.48	< 0.0001**
AB	307	1	307	1.56	0.2511
AC	3107.74	1	3107.74	15.84	0.0053**
BC	1010.19	1	1010.19	5.15	0.0575
A^**2**^	715.74	1	715.74	3.65	0.0978
B^2^	402.88	1	402.88	2.05	0.195
C^2^	21.58	1	21.58	0.11	0.7499
Residual	1373.33	7	196.19	–	–
Lack of fit	1372.18	3	457.39	1590.55	< 0.0001
Pure error	1.15	4	0.2876	–	–
Cor Total	44,484.37	16	–	–	–

“*” is significant (P < 0.05); “**" is highly significant (P < 0.01).

The energy loss coefficient reflects the ability of the flow stabilizer channel to dissipate the mechanical energy of the water flow, and the sensitivity of the flow rate to the pressure change reflects the flow stabilization performance of the flow stabilizer channel. From Eq. (12), it can be seen that when the *b* and *ρ* remain constant, the energy loss coefficient of the channel is directly proportional to the pressure difference ∆P and inversely proportional to the square of the flow rate. In other words, when the water flow with a constant pressure passes through the helical hedge flow channel, the smaller the outlet pressure, the greater the pressure difference between the inlet and outlet. This indicates that the energy loss coefficient of the channel is larger, and the flow rate decreases after energy dissipation, indicating a larger energy loss coefficient of the channel. Therefore, the energy loss of the channel under different parameter combinations can be determined by the pressure drop and flow velocity.

From Eq. (13), it can be seen that when the *b* and *ρ* remain constant, the larger the energy loss coefficient, the less the flow rate is affected by the pressure change. In other words, the sensitivity of the flow rate to the pressure change is lower, indicating better flow stabilization performance. This is consistent with the research conclusions of Qing ^22^. Understanding the energy loss of the helical hedge flow channel is of great significance for optimizing the hydraulic performance of the channel and improving the uniformity of water distribution for this type of channel emitter. The novel channel proposed in this study displays high energy loss characteristics. However, it remains unclear whether the channel possesses good anti-clogging capabilities due to the presence of multiple corners. Further exploration and research are necessary to investigate this aspect.

## Conclusion

**Figure 7:**
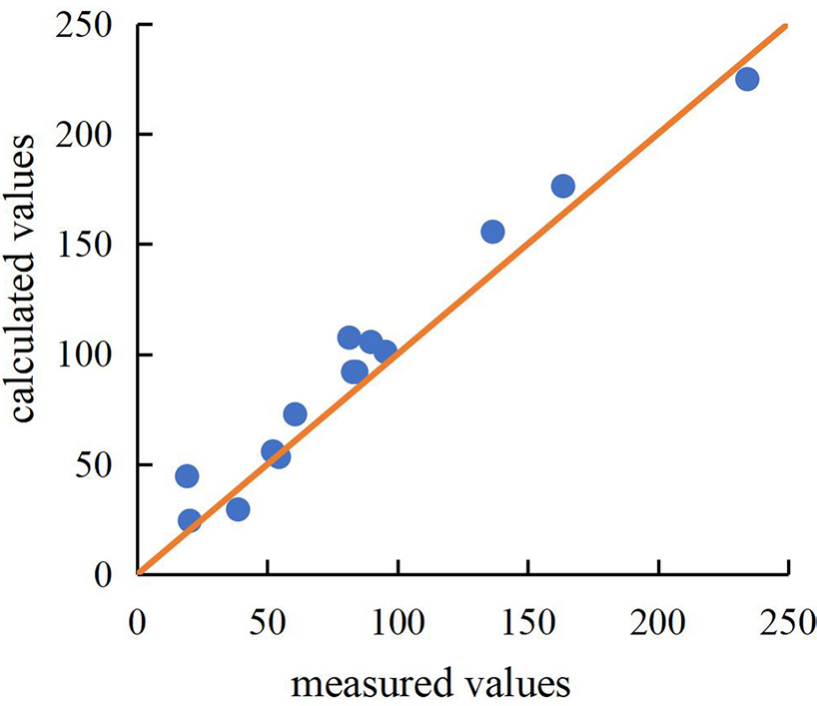
The comparison between the measured and calculated values of the energy loss coefficient.

The flow through the Tesla valve channel results in significant energy losses. However, this type of channel has not yet been utilized in emitters, Initial experiments have indicated that emitter incorporating this channel type exhibit superior hydraulic performance. Nevertheless, the impact of different channel parameters on flow energy losses remains unclear. In this study, we conducted research on the new channel using single-factor experiments and response surface experiments. We identified the primary channel parameters that influence the energy loss coefficient and determined that local head loss is the primary form of energy loss in the channel (with head loss along the path accounting for only 0.06–0.47% of the local head loss). We established a relationship between the main influencing parameters and the energy loss coefficient (R^2^ = 0.9691). By comparing measured and calculated values (Fig. 7), we observed that Eq. (20) accurately predicts the energy loss coefficient of the channel. The findings of this study can serve as decision-making references for the analysis and design optimization of energy loss in emitter incorporating this type of channel.

## Data Availability

The data that support the findings of this study are available from the corresponding author on reasonable request.
